# An interaction between fetal sex and placental weight and efficiency predicts intrauterine growth in response to maternal protein insufficiency and gestational exposure window in a mouse model of FASD

**DOI:** 10.1186/s13293-020-00320-9

**Published:** 2020-07-20

**Authors:** Sze Ting Cecilia Kwan, Brandon H. Presswood, Kaylee K. Helfrich, Joshua W. Baulch, Sandra M. Mooney, Susan M. Smith

**Affiliations:** 1 Nutrition Research Institute, University of North Carolina at Chapel Hill, Room #3111, 500 Laureate Way, Kannapolis, NC 28081 USA; 2 Nutrition Research Institute, Department of Nutrition, University of North Carolina at Chapel Hill, Room #3104, 500 Laureate Way, Kannapolis, NC 28081 USA

**Keywords:** Fetal stress response, Prenatal alcohol exposure, Maternal protein insufficiency, Fetal sex, Placental development, Intrauterine growth, Litter size, Fetal alcohol spectrum disorder

## Abstract

**Background:**

Individuals exposed to gestational stressors such as alcohol exhibit a spectrum of growth patterns, suggesting individualized responses to the stressors. We hypothesized that intrauterine growth responses to gestational alcohol are modified not only by the stressor’s severity but by fetal sex and the placenta’s adaptive capacity.

**Methods:**

Pregnant C57BL/6J mice were assigned to one of three groups. Group 1 consumed a normal protein diet (18% protein by weight) and received 4.5 g alcohol/kg body weight (NP-Alc-8) or isocaloric maltodextrin (NP-MD-8) daily from embryonic day (E) 8.5–E17.5. Group 2 consumed the same diet but received alcohol (NP-Alc-13) or maltodextrin (NP-MD-13) daily from E13.5–E17.5. Group 3 consumed the same diet but containing a lower protein content (12% protein by weight) from E0.5 and also received alcohol (LP-Alc-8) or maltodextrin (LP-MD-8) daily from E8.5–E17.5. Maternal, placental, and fetal outcomes were assessed on E17.5 using 2-way ANOVA or mixed linear model.

**Results:**

We found that intrauterine growth differed in the alcohol-exposed fetuses depending on sex and insult severity. Both NP-Alc-8 (vs. NP-MD-8) males and females had lower body weight and asymmetrical growth, but only NP-Alc-8 females had lower placental weight (*P* < 0.05). NP-Alc-13 (vs. NP-MD-13) females, but not their male littermates, had lower body weight (*P* = 0.019). Alcohol exposure beginning from E8.5 (vs. E13.5) decreased the ratio of fetal liver-to-body weight and increased the ratio of fetal brain-to-liver weight in both sexes (*P* < 0.05). LP-Alc-8 (vs. NP-MD-8) group had smaller litter size (*P* = 0.048), but the survivors had normal placental and body weight at E17.5. Nevertheless, LP-Alc-8 fetuses still showed asymmetrical growth. Correlation analyses reveal a relationship between litter size and placental outcomes, which were related to fetal outcomes in a sex-dependent manner, suggesting that the placenta may mediate the consequence of LP-Alc-altered litter size on fetal development.

**Conclusions:**

Our data indicate that the placenta is strongly involved in the fetal stress response and adapts in a sex-dependent fashion to support fetal development under the alcohol stressor. These variables may further influence the spectrum of intrauterine growth outcomes observed in those diagnosed with fetal alcohol spectrum disorder.

## Background

In the 1980s, British epidemiologist Dr. David Barker proposed that the prenatal period has a critical influence upon the individual’s lifelong health. According to the Developmental Origins of Health and Disease (DOHaD) hypothesis, adverse insults in the prenatal period alter normal fetal development and cause intrauterine growth restriction, which is a risk factor for cardiovascular and metabolic diseases in later life [1]. Gestational alcohol exposure is one common stressor encountered by the developing fetus. Twelve percent of pregnant women in the USA continue drinking and 4% practice binge drinking during their pregnancy [2]; the prevalence of maternal drinking during pregnancy is even higher in some European countries and can approach 40 to 60% [3]. Gestational alcohol exposure can lead to prenatal and postnatal growth deficits, structural birth defects, and neuroanatomical changes that cause lifelong neurocognitive impairments; these are collectively termed fetal alcohol spectrum disorder (FASD). These abnormalities are not independent from each other, and growth pattern is predictive of postnatal cognitive performance [4]. FASD affects approximately 5% of the first graders in the USA [5] and as many as 11% of children in populations where maternal alcohol abuse is widespread [6]. Globally, FASD represents a leading preventable cause of lifelong developmental and intellectual disability.

The developmental outcomes in children affected with FASD vary widely, suggesting that fetal responses to the alcohol-induced suboptimal intrauterine environment differ. Differences in maternal drinking pattern, alcohol dosage, and exposure window are obvious influencers upon the intrauterine experience, and thus, alter the fetal responses and developmental trajectory [7]. Indeed, clinical data confirm that heavy maternal binge drinking throughout pregnancy produces the worst growth and developmental impairments in alcohol-exposed children [8, 9]. This may represent one severe end of the stress spectrum, and the fetal response to this insult likely differs from that to a less severe insult.

The imposition of additional stressors, such as poor maternal nutritional status or lack of access to health care, could limit the fetus’s ability to adapt to the initial alcohol stressor and thus worsen the severity of alcohol’s impact. This could help explain how higher maternal socioeconomic status offers an apparent “buffering” against alcohol-mediated fetal damage [10–13]. Conversely, poor iron status is associated with worse growth outcomes in infants with FASD [14, 15], in part because alcohol interacts with maternal iron deficiency to cause anemia and reduce both prenatal and postnatal growth [16, 17]. Similarly, FASD rates are exceptionally high in an alcohol-abusing population in the Western Cape Province of South Africa [18] in which the pregnant mothers also consume significantly less protein than the dietary recommendation [19, 20]. Although these secondary stressors are commonplace, their impact on FASD is incompletely understood.

Investigations of other adverse intrauterine perturbations report a sexually dimorphic fetal response to the stressors [21]. Although both alcohol-exposed males and females similarly exhibit the hallmark features defining FASD, fetal sex appears to have an additional modulatory influence upon these outcomes. For example, Qazi and Masakawa note that their cohort of children diagnosed with FASD consists of more females than males [22]. Likewise, May and colleagues [23] recently reported that maternal drinking increases the males’ risk of mortality prior to birth but causes more growth impairments in females compared to their surviving male counterparts. The sex-specific effects of gestational alcohol exposure are also evidenced in the children’s dysregulated activation of the hypothalamic-pituitary-gonadal and hypothalamic-pituitary-adrenal axes as well as altered production of reproductive hormones and inflammatory markers [24–26].

We have a limited understanding of how secondary stressors interact with gestational alcohol exposure to alter fetal growth and know even less about how the fetus adapts—or fails to adapt—its response to the stressor. Evidence from investigating other adverse prenatal stressors suggests that the fetal responses begin in the placenta [21]. Positioned between the mother and the fetus, the placenta functions as an intrauterine sensor [27, 28] and responds to the adverse insult by modulating its morphology and functional capacity in order to enhance nutrient delivery to the fetus. Variations in the placental nutrient supply have lasting impacts on fetal development, and may contribute to different growth outcomes and confer differential risk of disease development [29, 30]. Whether the placenta is similarly involved in fetal responses to the alcohol insult remains to be determined. Here, we generated an animal model of FASD with different insult severity to examine how the alcohol-exposed fetus responds to these stressors. We hypothesize that the placenta plays a critical role in the fetal responses to the alcohol insult. Furthermore, we predict that the responses differ depending upon the stress severity and upon the sex of the alcohol-exposed fetus.

## Methods

### Animals, diets, and alcohol administration

Animal protocols were approved by the Institutional Animal Care and Use Committee at the David H. Murdock Research Institute (DHMRI) and were performed in accordance with the US Guide for the Care and Use of Laboratory Animals. Five-week-old male and female C57BL/6J mice were purchased from The Jackson Laboratory (Bar Harbor, ME) and were housed in a temperature-controlled room with a 12-h light/dark cycle at the AAALAC-accredited DHMRI Center for Laboratory Animal Science facilities. The mice were provided ad libitum access to water and the standard AIN-93G diet (normal protein, NP), which contains 18% protein by weight ([31]; TD. 94045; Envigo Teklad, Madison, WI). Females were mated at 8–9 weeks old. The day a vaginal plug was found was defined as embryonic day (E) 0.5.

On E0.5, dams were assigned into one of three groups with different alcohol insult severity. Dams in Group 1 (*n* = 20) experienced a moderate stressor. They continued the NP diet and received by gavage 4.5 g alcohol/kg body weight (200 proof alcohol, USP grade; Decon Labs, King of Prussia, PA) daily as two half-doses 1.5 h apart (NP-Alc-8, *n* = 11) or isocalorically equivalent maltodextrin (NP-MD-8, *n* = 9) beginning from E8.5 through E17.5. This high dose, binge alcohol exposure was selected to induce fetal growth deficits and included both the period of placental morphological and vascular development and the period of rapid fetal growth and increased nutrient demand [32–34]. Dams in Group 2 (*n* = 19) experienced a milder stressor; they also consumed the NP diet and received the same alcohol (NP-Alc-13, *n* = 10) and maltodextrin (NP-MD-13, *n* = 9) dose as above but only from E13.5 through E17.5. This shorter exposure period targeted the rapid fetal growth and increased nutrient demand. Dams in Group 3 (*n* = 22) received a severe stressor, which included a low protein diet (LP) that was compositionally identical to the NP diet except that it contained 12% protein by weight, with the caloric difference made up with cornstarch (TD. 190098; Envigo Teklad, Madison, WI). This protein level modeled the protein consumption of pregnant women in South African FASD cohorts relative to the dietary recommendation [18–20]. Dams in Group 3 also received the same daily alcohol (LP-Alc-8, *n* = 13) or maltodextrin (LP-MD-8, *n* = 9) gavage as Group 1 from E8.5 through E17.5.

**Fig. 1:**
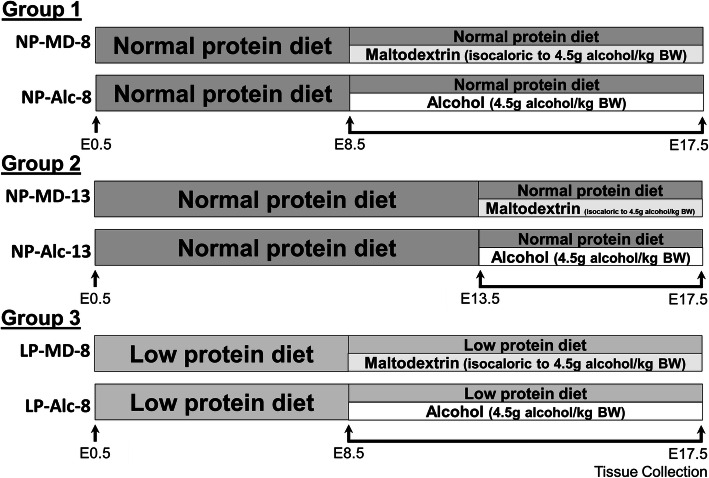
The overall experimental design. The first analysis examines the impacts of gestational alcohol exposure beginning from embryonic day (E) 8.5 to E17.5 (Group 1). The second analysis addresses the impacts of shortening the alcohol exposure window from E8.5–E17.5 (Group 1) to E13.5–E17.5 (Group 2). The third analysis investigates the effects of inadequate maternal protein intake in alcohol-drinking dams (Group 1 vs. Group 3). Abbreviations: Alc, alcohol; BW, body weight; LP, low protein; MD, maltodextrin; NP, normal protein

Dams were weighed on E0.5 and then daily throughout the alcohol (or maltodextrin) exposure period. Maternal food consumption was recorded as the total amount of food consumed from E0.5 through E17.5. The study design summarizing the diet and alcohol administration is shown in Fig. 1.

### Blood alcohol concentration analysis

Blood alcohol concentrations (BACs) were determined using a separate group of pregnant mice fed the NP diet (*n* = 3). Blood was drawn from the saphenous vein 30 min after the second alcohol gavage on E8.5 to quantify peak BAC [35, 36]. BAC analysis was performed using the Analox instruments GM7 Micro-Stat following the manufacturer’s protocol. Briefly, whole blood sample was precipitated with 6% perchloric acid and then neutralized with 1.5 M K_2_CO_3_. The mixture was centrifuged at 20,000 rpm for 3 min, and the supernatant was used for analysis. Maternal BAC at 30 min after the second gavage was 217.3 ± 20.8 mg/dL.

### Tissue collection and fetal anthropometry assessment

On E17.5, 2 h after the second gavage, dams were euthanized by an overdose of isoflurane. The uterine horn was removed and weighed. The number and position of surviving fetuses and fetal resorptions were recorded. The placentas were isolated and weighed after the maternal decidua was trimmed and removed. The fetuses were isolated and weighed. The fetal brain, liver, and heart were then dissected and weighed. The fetal tail was collected for determining the sex of the fetus.

### Fetal sex genotyping

A clip of fetal tail was added to 600 μL genomic lysis buffer (20 mM Tris-HCl, 150 mM NaCl, 100 mM EDTA, 1% SDS) plus 3 μL 20 mg/mL proteinase K and was incubated at 55 °C overnight. The following morning, protein precipitation solution (200 μL of A7953, Promega, Madison, WI) was added to the sample, followed by centrifugation at 14,500×*g* for 3 min. The supernatant containing DNA was then mixed with 600 μL isopropanol and centrifuged at 14,500×*g* for 1 min. The DNA pellet was then washed with 70% ethanol, air-dried, and resuspended in 30 μL TE buffer. DNA concentration was determined using Nanodrop. Approximately 600 ng/μL DNA was used in PCR reaction to determine fetal sex. The primers for the *Sry* gene were F: 5′-TGGGACTGGTGACAATTGTC-3′, R: 5′-GAGTACAGGTGTGCAGCTCT-3′. The autosomal *Il3* gene was used to serve as an internal control for the female genome, and its primers were F: 5′-GGGACTCCAAGCTTCAATCA-3′, R: 5′-TGGAGGAGGAAGAAAAGCAA-3′ [37]. All primers were purchased from Integrated DNA Technologies (Coralville, IA). The PCR procedure was performed using a commercial kit (Invitrogen, Carlsbad, CA) according to the manufacturer’s protocol. The PCR products were resolved by electrophoresis at 100 V for 30 min in 2% agarose gels containing ethidium bromide. The bands (402 bp for the *Sry* amplicon and 544 bp for the *Il3* amplicon) were visualized using the Azure Biosystems (Neobits Inc, Santa Clara, CA).

### Statistical analysis

All data were checked for normality by visual examination of the histogram and by the Shapiro-Wilk Test and for equal variance by visual examination of the residual plot and by the Levene’s test. If one of these assumptions was not met, data were natural-logarithmically transformed. Intergroup comparisons of the maternal and litter outcomes were analyzed using two-way ANOVA, followed by pairwise comparisons with Benjamini-Hochberg method applied to correct for multiple testing. For the comparison of exposure length (Group 1 versus 2), the ANOVA model included alcohol treatment, timing of exposure, and their interaction as factors. For the comparison of maternal protein intake (Group 1 versus 3), the ANOVA model included diet treatment, alcohol treatment, and their interaction as factors. The placental and fetal outcomes were analyzed separately for males and females using mixed linear model including alcohol treatment, exposure length (for Group 1 versus 2), diet treatment (for Group 1 versus 3), and their interactions as independent fixed effect factors, and litter as an independent random effect factor. Post hoc pairwise comparisons were performed using the Benjamini-Hochberg procedure to correct for multiple testing. For all analyses, litter size was included in the models as a covariate if its *P* value ≤ 0.05. For data that still could not meet the assumptions after natural-log transformation, Kruskal-Wallis test was used, followed by pairwise *t* test comparisons with Benjamini-Hochberg correction. These analyses were performed using SPSS, Version 26 (SPSS Inc, Chicago, IL). Because placental and fetal outcomes differed between males and females, the correlation analyses were performed separately for each fetal sex using the *ggpubr* package under the R software, Version 3.4.3 [38]. Benjamini-Hochberg correction was applied to adjust for multiple comparisons. Data are shown as means ± SEM. Differences were considered as statistically significant at *P* < 0.05, and *P* < 0.10 was indicative of trends.

## Results

### Moderate stressor: gestational alcohol exposure from E8.5 to E17.5

#### Pregnancy outcomes

**Fig. 2:**
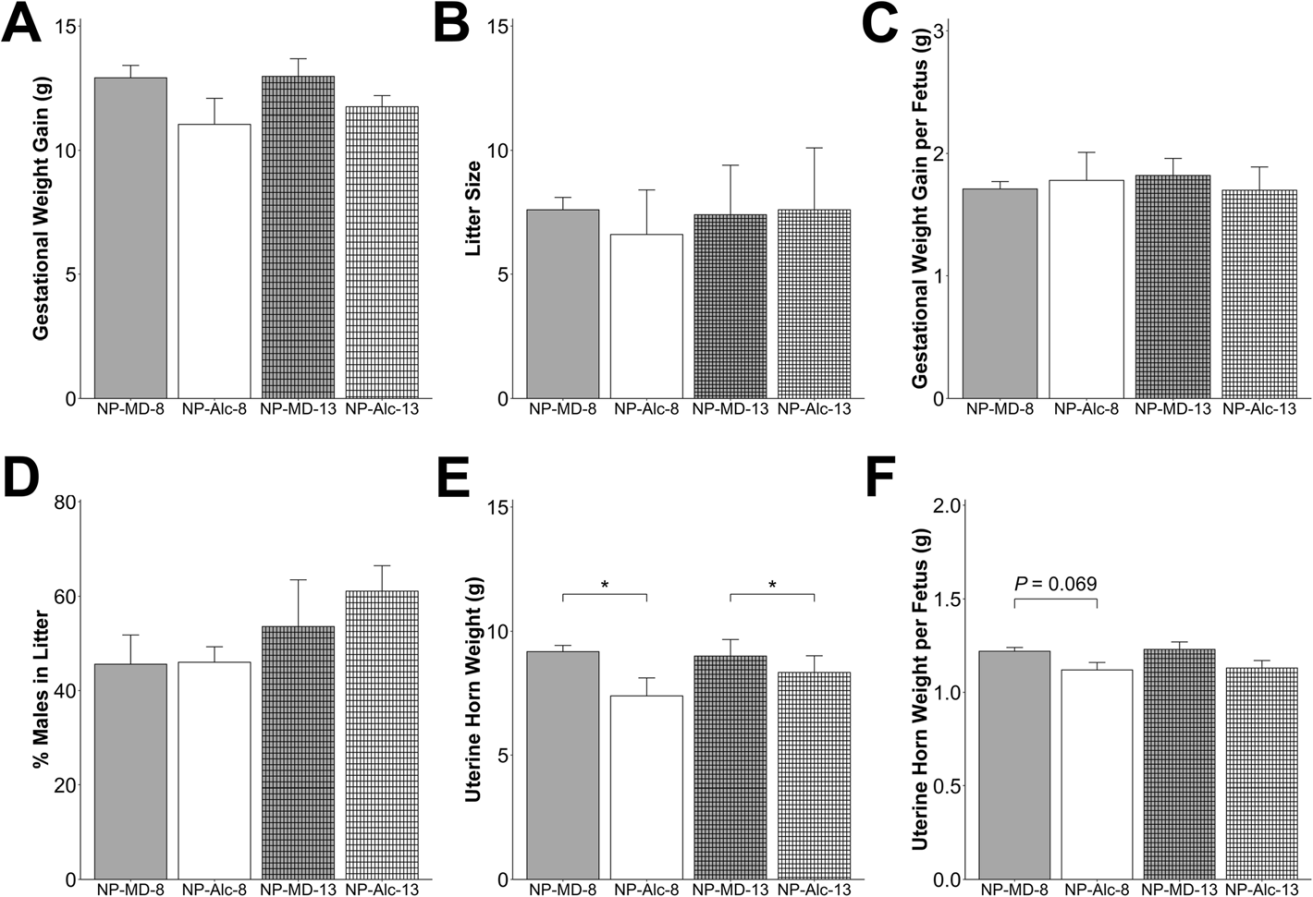
Gestational alcohol exposure beginning from embryonic day (E) 8.5 to E17.5 and E13.5 to E17.5 and comparisons of these exposure windows with respect to gestational weight gain (**a**), litter size (**b**), gestational weight gain per fetus (**c**), percent males in litter (**d**), uterine horn weight (**e**), and uterine horn weight per fetus (**f**). Data were analyzed using two-way ANOVA, and post hoc pairwise comparisons were analyzed using pairwise *t* test with Benjamini-Hochberg correction. Values are presented as means ± SEMs (**a**, **c**–**f**) or mean ± SD (**b**). *n* = 9 dams for NP-MD-8 and NP-MD-13 groups, *n* = 8 dams for NP-Alc-8 group, and *n* = 10 dams for NP-Alc-13 group. Asterisk (*) denotes statistical significance (*P* < 0.05) for the indicated comparison. Abbreviations: Alc, alcohol; MD, maltodextrin; NP, normal protein

Alcohol exposure from E8.5 to E17.5 did not affect maternal body weight (Supplemental Table 1), food intake (Supplemental Table 1), gestational weight gain (Fig. 2a), litter size (Fig. 2b), gestational weight gain per fetus (Fig. 2c), or percent of males in the litter (Fig. 2d). This high dose, binge alcohol exposure resulted in three maternal deaths prior to E17.5. Alcohol exposure significantly reduced uterine horn weight (− 19%, *P* = 0.048; Fig. 2e), and uterine horn weight per fetus trended to be reduced (− 7%, *P* = 0.069; Fig. 2f).

#### Placental and fetal outcomes

**Fig. 3:**
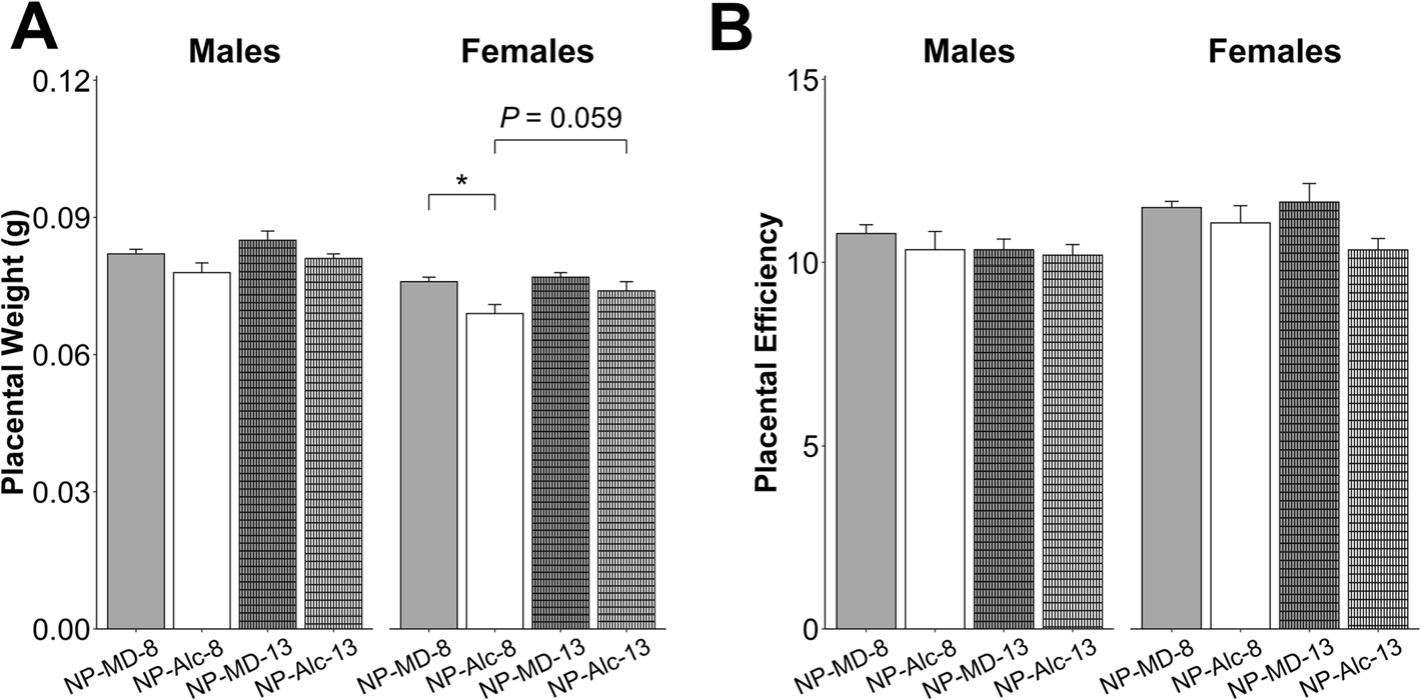
Gestational alcohol exposure beginning from embryonic day (E) 8.5 to E17.5 and E13.5 to E17.5 and comparisons of these exposure windows with respect to placental weight (**a**) and placental efficiency (**b**). Data were analyzed separately for males and females using mixed linear model followed by post hoc Benjamini-Hochberg corrections to adjust for multiple comparisons. Values are presented as means ± SEMs. *n* = 9 dams for NP-MD-8 and NP-MD-13 groups, *n* = 8 dams for NP-Alc-8 group, and *n* = 10 dams for NP-Alc-13 group. Asterisk (*) denotes statistical significance (*P* < 0.05) for the indicated comparison. Abbreviations: Alc, alcohol; MD, maltodextrin; NP, normal protein

Alcohol exposure from E8.5 to E17.5 decreased female, but not male, placental weight by 9% (*P* = 0.046; Fig. 3a) but did not affect placental efficiency for either sex (Fig. 3b).

**Fig. 4:**
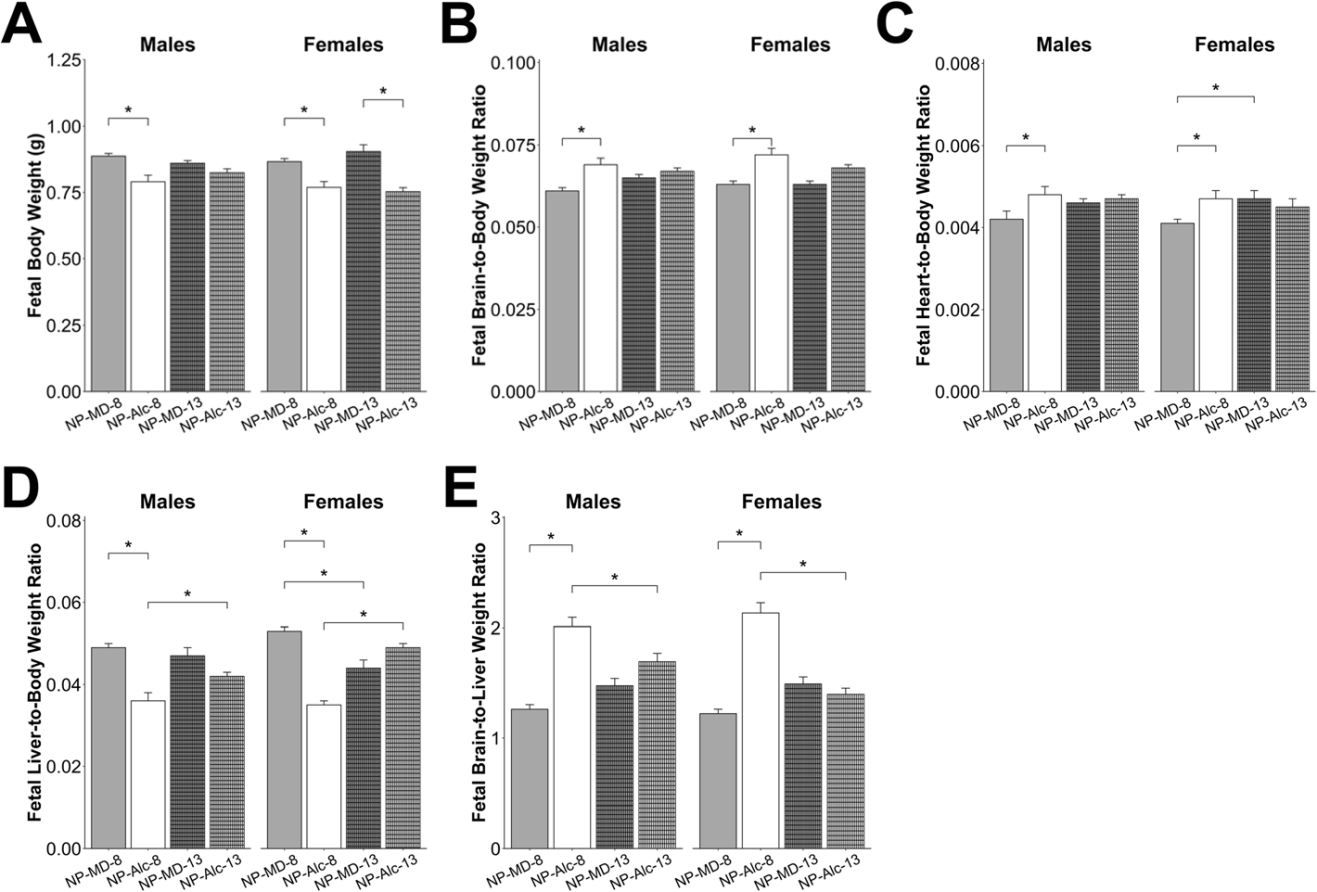
Gestational alcohol exposure beginning from embryonic day (E) 8.5 to E17.5 and E13.5 to E17.5 and comparisons of these exposure windows with respect to fetal body weight (**a**), fetal brain-to-body weight ratio (**b**), fetal heart-to-body weight ratio (**c**), fetal liver-to-body weight ratio (**d**), and fetal brain-to-liver weight ratio (**e**). Data were analyzed separately for males and females using mixed linear model followed by post hoc Benjamini-Hochberg corrections to adjust for multiple comparisons. Values are presented as means ± SEMs. *n* = 9 dams for NP-MD-8 and NP-MD-13 groups, *n* = 8 dams for NP-Alc-8 group, and *n* = 10 dams for NP-Alc-13 group. Asterisk (*) denotes statistical significance (*P* < 0.05) for the indicated comparison. Abbreviations: Alc, alcohol; MD, maltodextrin; NP, normal protein

In the male fetuses, the long-term alcohol exposure reduced body weight (− 11%, *P* = 0.023; Fig. 4a), liver weight (− 36%, *P* < 0.001; Supplemental Table 2), and liver-to-body weight ratio (− 27%, *P* < 0.001; Fig. 4d), and increased brain-to-body (+ 13%, *P* = 0.004; Fig. 4b), heart-to-body (+ 14%, *P* = 0.027; Fig. 4c), and brain-to-liver (+ 59%, *P* < 0.001; Fig. 4e) weight ratios; all these values suggested asymmetrical fetal growth.

The alcohol exposure had similar effects upon their female littermates, and it reduced female body weight (− 11%, *P* = 0.049; Fig. 4a), liver weight (− 41%, *P* < 0.001; Supplemental Table 2), and liver-to-body weight ratio (− 34%, *P* < 0.001; Fig. 4d), and it elevated brain-to-body (+ 14%, *P* = 0.004; Fig. 4b), heart-to-body (+ 15%, *P* = 0.038; Fig. 4c), and brain-to-liver weight ratios (+ 75%, *P* < 0.001; Fig. 4e).

Correlation analyses revealed that, for males, litter size was inversely related to placental weight (*R* = − 0.49, *P* = 0.008) and positively related to placental efficiency (*R* = 0.48, *P* = 0.008; Table 1). For females, litter size correlated only with placental efficiency (*R* = 0.39, *P* = 0.043) and not the placental weight (Table 1). Although none of the fetal anthropometric indices were associated with litter size (Table 1), they were related to placental weight and/or efficiency in a sex-dependent manner (Table 2). Specifically, for male fetuses, body weight was strongly correlated with placental efficiency (*R* = 0.82, *P* < 0.001), whereas brain-to-body weight ratio was negatively associated (*R* = − 0.67, *P* < 0.001). For female fetuses, body weight was positively related to placental efficiency (*R* = 0.55, *P* < 0.001) and placental weight (*R* = 0.48, *P* = 0.001). Additionally, their placental weights were also associated with brain-to-body (*R* = − 0.53, *P* < 0.001), heart-to-body (*R* = − 0.42, *P* = 0.003), liver-to-body (*R* = 0.41, *P* = 0.004), and brain-to-liver weight ratios (*R* = − 0.48, *P* = 0.001).

**Table 1:** Correlation of the anthropometric measurements of the placentas and fetuses from dams in Group 1 with their litter size

	Males only			Females only		
	*R*	*P*_unadjusted_	*P*_BH-adjusted_	*R*	*P*_unadjusted_	*P*_BH-adjusted_
Placental weight	− 0.49	0.002	0.008*	− 0.18	0.22	0.39
Placental efficiency	0.48	0.002	0.008*	0.39	0.006	0.043*
Fetal body weight	0.28	0.10	0.22	0.22	0.13	0.35
Fetal brain-to-body weight ratio	− 0.17	0.31	0.54	− 0.21	0.15	0.35
Fetal heart-to-body weight ratio	0.02	0.91	0.94	− 0.002	0.99	0.99
Fetal liver-to-body weight ratio	− 0.01	0.94	0.94	0.14	0.33	0.39
Fetal brain-to-liver weight ratio	0.02	0.90	0.94	− 0.15	0.29	0.39

Litters from both treatments were combined and included in the analyses. The sample sizes were *n* = 9 litters for NP-MD-8 group; *n* = 8 litters for NP-Alc-8 group. *P* values before and after multiple testing adjustment using Benjamini-Hochberg method are presented

*Statistical significance at *P* < 0.05

**Table 2:** Correlation of the anthropometric measurements of the fetuses from dams in Group 1 with their placental weight and efficiency

	Correlation with placental weight			Correlation with placental efficiency		
	*R*	*P*_unadjusted_	*P*_BH-adjusted_	*R*	*P*_unadjusted_	*P*_BH-adjusted_
Males only						
Fetal body weight	− 0.001	0.99	0.99	0.82	< 0.001	< 0.001*
Fetal brain-to-body weight ratio	0.15	0.38	0.63	− 0.67	< 0.001	< 0.001*
Fetal heart-to-body weight ratio	− 0.04	0.81	0.99	− 0.17	0.31	0.52
Fetal liver-to-body weight ratio	0.26	0.11	0.35	− 0.07	0.68	0.76
Fetal brain-to-liver weight ratio	− 0.24	0.14	0.35	− 0.05	0.76	0.76
Females only						
Fetal body weight	0.48	< 0.001	0.001*	0.55	< 0.001	< 0.001*
Fetal brain-to-body weight ratio	− 0.53	< 0.001	< 0.001*	− 0.16	0.28	0.70
Fetal heart-to-body weight ratio	− 0.42	0.003	0.003*	0.04	0.78	0.84
Fetal liver-to-body weight ratio	0.41	0.004	0.004*	− 0.05	0.72	0.84
Fetal brain-to-liver weight ratio	− 0.48	< 0.001	0.001*	0.03	0.84	0.84

Litters from both treatments were combined and included in the analyses. The sample sizes were *n* = 9 litters for NP-MD-8 group; *n* = 8 litters for NP-Alc-8 group. *P* values before and after multiple testing adjustment using Benjamini-Hochberg method are presented

*Statistical significance at *P* < 0.05

### Mild stressor: shorter gestational alcohol exposure window, from E13.5 to E17.5

#### Pregnancy outcomes

For the shorter alcohol exposure from E13.5 to E17.5, alcohol did not affect maternal body weight (Supplemental Table 1), food intake (Supplemental Table 1), gestational weight gain (Fig. 2a), litter size (Fig. 2b), gestational weight gain per fetus (Fig. 2c), or percent of males in the litter (Fig. 2d). Alcohol exposure significantly reduced uterine horn weight (− 7%, *P* = 0.050; Fig. 2e) and trended to reduce uterine horn weight per fetus (*P* = 0.094; Fig. 2f). None of these outcomes differed in the secondary comparison between the NP-Alc-8 and NP-Alc-13 groups or between the NP-MD-8 and NP-MD-13 groups.

#### Placental and fetal outcomes

Alcohol exposure from E13.5 to E17.5 did not affect placental weight (Fig. 3a) or placental efficiency (Fig. 3b) for either sex. In male fetuses, alcohol exposure reduced liver weight (− 15%, *P* = 0.017; Supplemental Table 2) but not body weight, brain weight, heart weight, nor the ratios of these organ weights to body weight (Fig. 4a–e, Supplemental Table 2). In contrast to their male littermates, alcohol exposure reduced female body weight by 17% (*P* = 0.019; Fig. 4a) and trended to reduce their heart weight by 20% (*P* = 0.072; Supplemental Table 2). No other differences were found in these females.

When we compared fetal growth outcomes in the E8.5–E17.5 and E13.5–E17.5 exposures, we found that the duration of alcohol exposure affected these outcomes in a sex-dependent manner, and the females were more impacted. Although male placental weight did not differ by alcohol exposure duration, the female placentas trended to be smaller when alcohol exposure began at E8.5 instead of E13.5 (− 7%, *P* = 0.059; Fig. 3a). However, both sexes in the NP-Alc-8 (vs. NP-Alc-13) group had a lower liver-to-body weight ratio (males: − 14%, *P* = 0.046; females: − 29%; *P* < 0.001; Fig. 4d) and a higher brain-to-liver weight ratio (males: + 19%, *P* = 0.022; females: + 53%; *P* < 0.001; Fig. 4e).

Although correlation analyses found no relationship between litter size and placental or fetal weights (Table 3), several associations emerged with placental weight and efficiency (Table 4), and these associations again were sex-dependent. For males, body weight (*R* = 0.70, *P* < 0.001) was positively associated with placental efficiency, whereas the brain-to-body (*R* = − 0.43, *P* < 0.001) and heart-to-body weight ratios (*R* = − 0.24, *P* = 0.035) were negatively associated. For females, similar relationships between placental efficiency, body weight (*R* = 0.75, *P* < 0.001), and the brain-to-body weight ratio (*R* = − 0.26, *P* = 0.027) were found. Additionally, female placental efficiency inversely correlated with liver-to-body weight ratio (*R* = − 0.25, *P* = 0.027), and placental weight was correlated to body weight (*R* = 0.27, *P* = 0.011), brain-to-body (*R* = − 0.35, *P* = 0.002), heart-to-body (*R* = − 0.31, *P* = 0.006), liver-to-body (*R* = 0.28, *P* = 0.009), and brain-to-liver weight ratios (*R* = − 0.39, *P* = 0.001).

**Table 3:** Correlation of the anthropometric measurements of the placentas and fetuses from dams in Groups 1 and 2 with their litter size

	Males only			Females only		
	*R*	*P*_unadjusted_	*P*_BH-adjusted_	*R*	*P*_unadjusted_	*P*_BH-adjusted_
Placental weight	− 0.05	0.65	0.92	− 0.11	0.29	0.46
Placental efficiency	0.06	0.57	0.92	− 0.02	0.84	0.88
Fetal body weight	0.03	0.73	0.92	− 0.09	0.33	0.46
Fetal brain-to-body weight ratio	0.01	0.93	0.93	− 0.15	0.10	0.33
Fetal heart-to-body weight ratio	0.02	0.79	0.92	− 0.01	0.88	0.88
Fetal liver-to-body weight ratio	− 0.15	0.09	0.53	0.13	0.14	0.33
Fetal brain-to-liver weight ratio	0.12	0.15	0.53	− 0.16	0.07	0.33

Litters from all 4 treatments were combined and included in the analyses. The sample sizes were *n* = 9 litters for NP-MD-8 and NP-MD-13 groups; *n* = 8 litters for NP-Alc-8 group; *n* = 10 litters for NP-Alc-13 group. *P* values before and after multiple testing adjustment using Benjamini-Hochberg method are presented

**Table 4:** Correlation of the anthropometric measurements of the fetuses from dams in Groups 1 and 2 with their placental weight and efficiency

	Correlation with placental weight			Correlation with placental efficiency		
	*R*	*P*_unadjusted_	*P*_BH-adjusted_	*R*	*P*_unadjusted_	*P*_BH-adjusted_
Males only						
Fetal body weight	0.12	0.24	0.40	0.70	< 0.001	< 0.001*
Fetal brain-to-body weight ratio	0.003	0.98	0.98	− 0.43	< 0.001	< 0.001*
Fetal heart-to-body weight ratio	− 0.01	0.91	0.98	− 0.24	0.021	0.035*
Fetal liver-to-body weight ratio	0.14	0.17	0.40	− 0.21	0.040	0.050
Fetal brain-to-liver weight ratio	− 0.17	0.09	0.40	0.09	0.36	0.36
Females only						
Fetal body weight	0.27	0.011	0.011*	0.75	< 0.001	< 0.001*
Fetal brain-to-body weight ratio	− 0.35	< 0.001	0.002*	− 0.26	0.012	0.027*
Fetal heart-to-body weight ratio	− 0.31	0.004	0.006*	0.05	0.62	0.62
Fetal liver-to-body weight ratio	0.28	0.007	0.009*	− 0.25	0.016	0.027*
Fetal brain-to-liver weight ratio	− 0.39	< 0.001	0.001*	0.14	0.19	0.24

Litters from all 4 treatments were combined and included in the analyses. The sample sizes were *n* = 9 litters for NP-MD-8 and NP-MD-13 groups; *n* = 8 litters for NP-Alc-8 group; *n* = 10 litters for NP-Alc-13 group. *P* values before and after multiple testing adjustment using Benjamini-Hochberg method are presented

*Statistical significance at *P* < 0.05

### Severe stressor: co-exposure to gestational alcohol and maternal protein insufficiency

#### Pregnancy outcomes

**Fig. 5:**
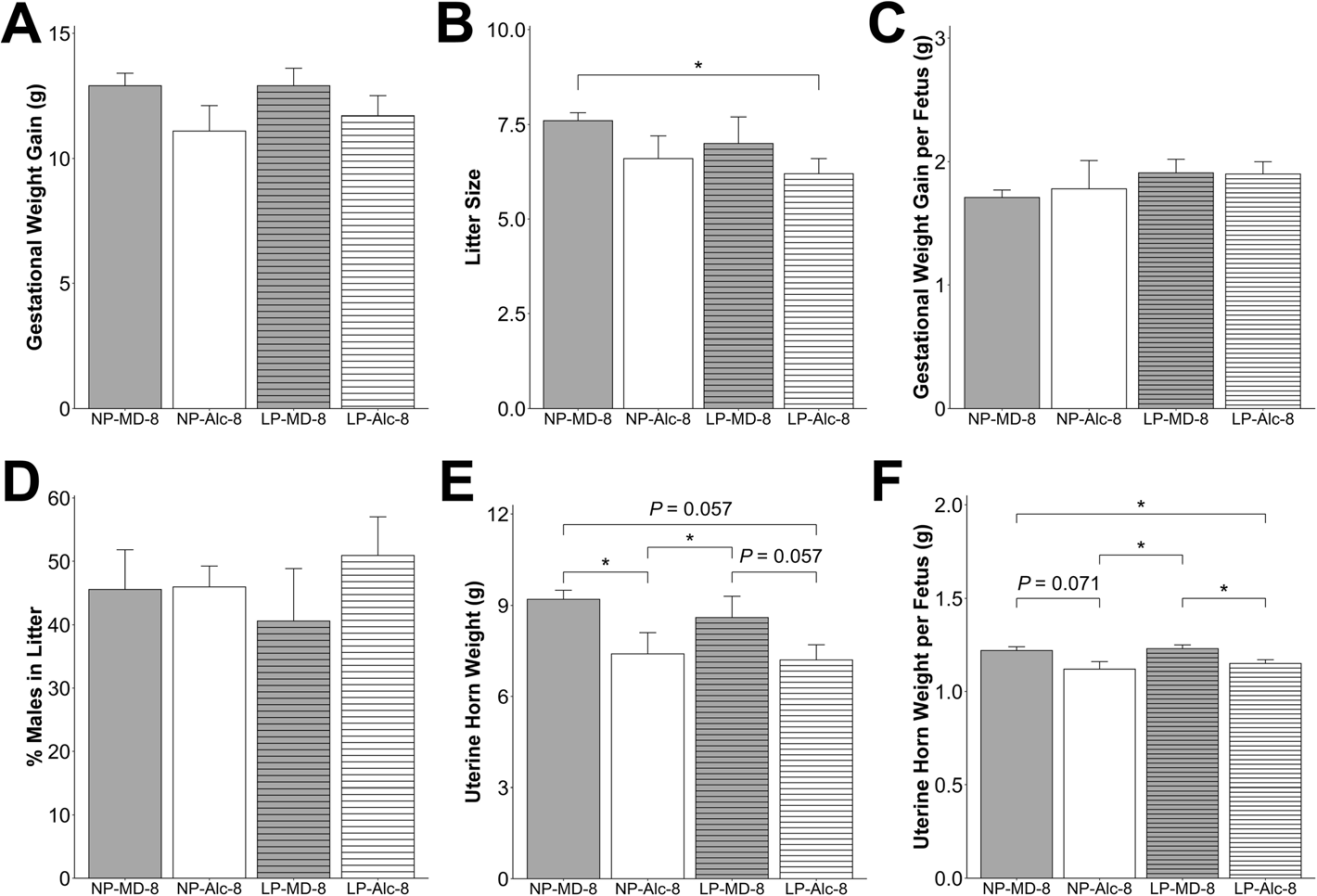
Effect of gestational alcohol exposure and maternal protein intake on gestational weight gain (**a**), litter size (**b**), gestational weight gain per fetus (**c**), percent males in litter (**d**), uterine horn weight (**e**), and uterine horn weight per fetus (**f**). Data for the NP-MD-8 and NP-Alc-8 groups are from Fig. 2 and are presented here for comparison purposes. Data were analyzed using two-way ANOVA, and post hoc pairwise comparisons were analyzed using pairwise *t* test with Benjamini-Hochberg correction. Values are presented as means ± SEMs (**a**, **c**–**f**) or mean ± SD (**b**). *n* = 9 dams for NP-MD-8, LP-MD-8, and LP-Alc-8 groups, and *n* = 8 dams for NP-Alc-8 group. Asterisk (*) denotes statistical significance (*P* < 0.05) for the indicated comparison. Abbreviations: Alc, alcohol; LP, low protein; MD, maltodextrin; NP, normal protein

The combination of longer alcohol exposure (E8.5–E17.5) plus low protein diet did not significantly affect maternal body weight, food intake, gestation weight gain, weight gain per fetus, or percent of males in the litter (Fig. 5 and Supplemental Table 1). Four dams from the LP-Alc-8 group were found dead prior to E17.5, and the surviving LP-Alc-8 dams had a smaller (*P* = 0.048) litter size than the NP-MD-8 dams (Fig. 5b). LP itself did not affect uterine horn weight or uterine horn weight per fetus, whereas its combination with alcohol (LP-Alc-8) reduced uterine horn weight (vs. LP-MD-8, − 16%, *P* = 0.057; Fig. 5e) and uterine horn weight per fetus (vs. LP-MD-8, − 7%, *P* = 0.042; Fig. 5f). LP-Alc-8 (vs. NP-MD-8) also reduced uterine horn weight (− 22%, *P* = 0.057; Fig. 5e) and uterine horn weight per fetus (− 5%, *P* = 0.046; Fig. 5f). However, these outcomes did not differ between the LP-Alc-8 and NP-Alc-8 dams.

#### Placental and fetal outcomes

**Fig. 6:**
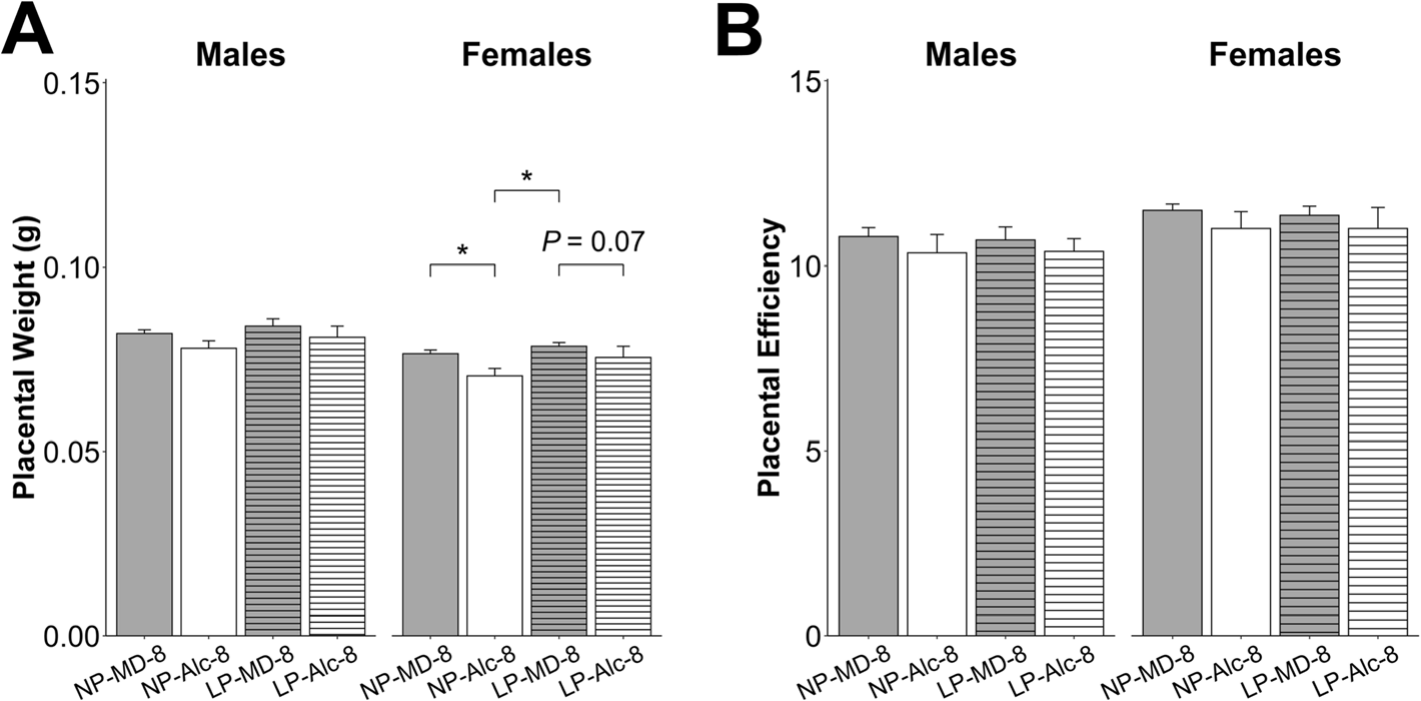
Effect of gestational alcohol exposure and maternal protein intake on placental weight (**a**) and placental efficiency (**b**). Data for the NP-MD-8 and NP-Alc-8 groups are from Fig. 3 and are presented here for comparison purposes. Data were analyzed separately for males and females using mixed linear model followed by post hoc Benjamini-Hochberg corrections to adjust for multiple comparisons. Values are presented as means ± SEMs. *n* = 9 litters for NP-MD-8, LP-MD-8, and LP-Alc-8 groups, and *n* = 8 litters for NP-Alc-8 group. Asterisk (*) denotes statistical significance (*P* < 0.05) for the indicated comparison. Abbreviations: Alc, alcohol; LP, low protein; MD, maltodextrin; NP, normal protein

Gestational alcohol exposure and maternal protein intake did not affect placental weight or efficiency in males (Fig. 6a, b). Females from the low-protein alcohol-exposed pregnancies showed a trend for reduced placental weight (vs. LP-MD-8, − 4%, *P* = 0.070; Fig. 6a), but placental efficiency was unaltered (*P* > 0.05; Fig. 6b).

**Fig. 7:**
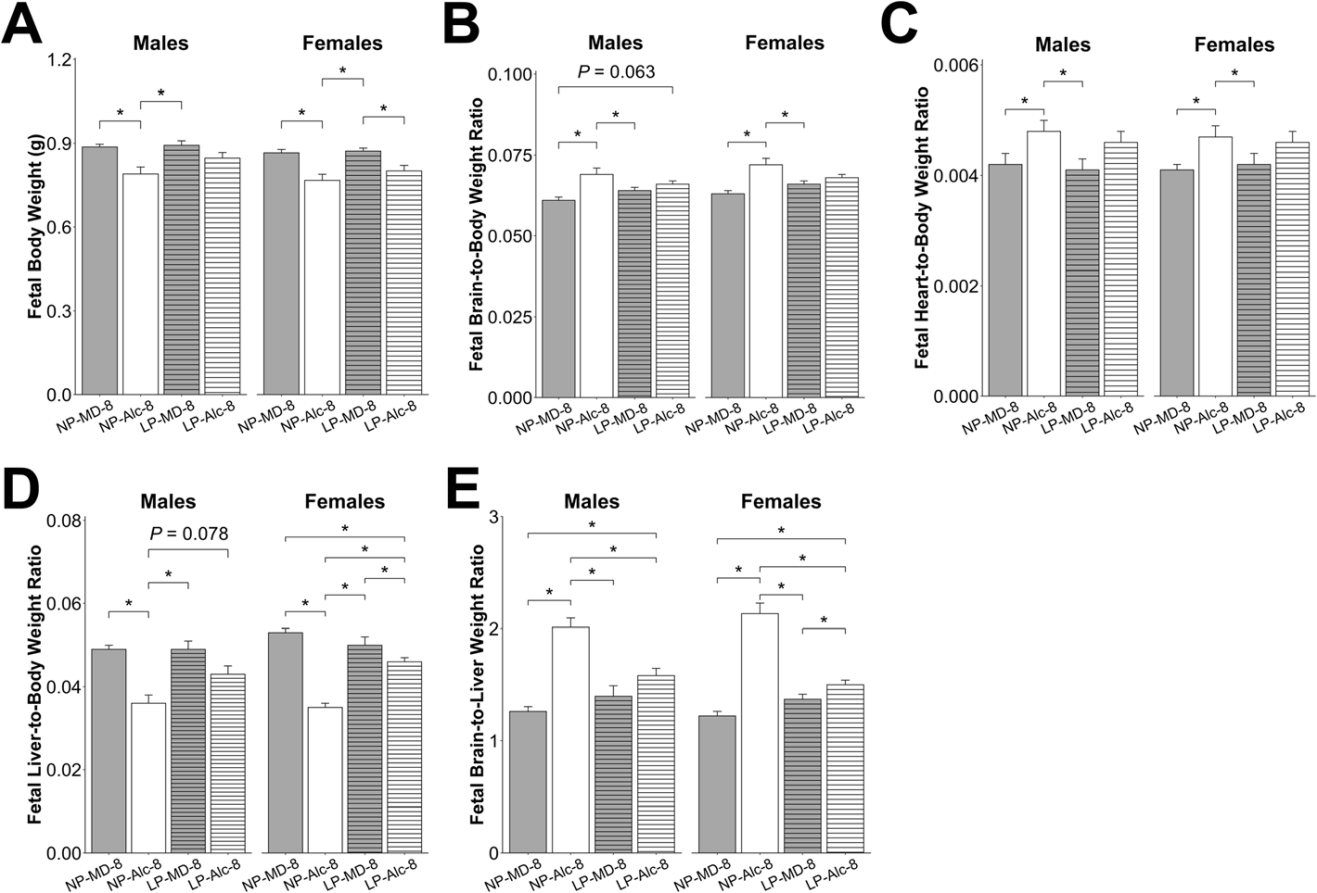
Effect of gestational alcohol exposure and maternal protein intake on fetal body weight (**a**), fetal brain-to-body weight ratio (**b**), fetal heart-to-body weight ratio (**c**), fetal liver-to-body weight ratio (**d**), and fetal brain-to-liver weight ratio (**e**). Data for the NP-MD-8 and NP-Alc-8 groups are from Fig. 4 and are presented here for comparison purposes. Data were analyzed separately for males and females using mixed linear model followed by post hoc Benjamini-Hochberg corrections to adjust for multiple comparisons. Values are presented as means ± SEMs. *n* = 9 litters for NP-MD-8, LP-MD-8, and LP-Alc-8 groups, and *n* = 8 litters for NP-Alc-8 group. Asterisk (*) denotes statistical significance (*P* < 0.05) for the indicated comparison. Abbreviations: Alc, alcohol; LP, low protein; MD, maltodextrin; NP, normal protein

With respect to fetal outcomes, alcohol exposure did not further affect the male fetuses in the low protein group with respect to body weight, organ weights, or organ-to-body weight ratios (Fig. 7a–d; Supplemental Table 2). These LP-Alc-8 males had a higher brain-to-liver weight ratio (+ 25%, *P* = 0.017; Fig. 7e) in the comparison against NP-MD-8 males. However, these ratios were even higher in the NP-Alc-8 males (vs. LP-Alc-8, + 27%, *P* = 0.017; vs. LP-MD-8, + 44%, *P* < 0.001; Fig. 7e).

In contrast to their male littermates, LP-Alc-8 females had a lower body weight compared to LP-MD-8 females (− 8%, *P* = 0.042; Fig. 7a) but not brain-to-body weight or heart-to-body weight ratios (Fig. 7b–c); these values also did not differ from those of NP-Alc-8 or NP-MD-8 females (Fig. 7a–c). The LP-Alc-8 females had a lower liver-to-body weight ratio compared with the LP-MD-8 (− 8%, *P* = 0.016) and NP-MD-8 (− 13%, *P* = 0.002) females (Fig. 7d). However, this ratio was the lowest in the NP-Alc-8 females (vs. LP-Alc-8, − 24%, *P* = 0.002; vs. LP-MD-8, − 30%, *P* < 0.001; Fig. 7d). As with their male littermates, alcohol exposure increased female brain-to-liver weight ratios in both the low protein (vs. LP-MD-8, + 10%, *P* = 0.018; vs. NP-MD-8, + 23%, *P* < 0.001) and normal protein pregnancies (vs. LP-Alc-8, + 41%, *P* < 0.001; vs. LP-MD-8, + 54%, *P* < 0.001), and this again suggested asymmetrical fetal growth (Fig. 7e).

Correlation analyses revealed, irrespective of fetal sex, an inverse relationship between litter size and placental weight (males: *R* = − 0.32, *P* = 0.030; females: *R* = − 0.27, *P* = 0.040) and a positive relationship between litter size and placental efficiency (males: *R* = 0.42, *P* < 0.001; females: *R* = 0.33, *P* < 0.001). No associations were detected between litter size and any of the fetal outcomes in males or females (Table 5). However, fetal anthropometric endpoints correlated with placental weight and/or placental efficiency in a sex-dependent manner (Table 6). For males, only body weight (*R* = 0.73, *P* < 0.001) and the brain-to-body weight ratio (*R* = − 0.55, *P* < 0.001) were associated with placental efficiency. Females displayed similar relationships of placental efficiency with body weight (*R* = 0.65, *P* < 0.001) and brain-to-body weight ratio (*R* = − 0.33, *P* = 0.002), plus an additional association of placental weight with liver-to-body (*R* = 0.44, *P* < 0.001) and brain-to-liver (*R* = − 0.42, *P* < 0.001) weight ratios.

**Table 5:** Correlation of the anthropometric measurements of the placentas and fetuses from dams in Groups 1 and 3 with their litter size

	Males only			Females only		
	*R*	*P*_unadjusted_	*P*_BH-adjusted_	*R*	*P*_unadjusted_	*P*_BH-adjusted_
Placental weight	− 0.32	0.006	0.030*	− 0.27	0.008	0.040*
Placental efficiency	0.42	< 0.001	< 0.001*	0.33	< 0.001	< 0.001*
Fetal body weight	0.24	0.040	0.13	0.19	0.058	0.18
Fetal brain-to-body weight ratio	− 0.10	0.39	0.65	− 0.17	0.11	0.22
Fetal heart-to-body weight ratio	0.05	0.67	0.74	− 0.03	0.81	0.81
Fetal liver-to-body weight ratio	− 0.07	0.57	0.74	− 0.18	0.073	0.18
Fetal brain-to-liver weight ratio	0.05	0.65	0.74	0.07	0.48	0.65

Litters from all 4 treatments were combined and included in the analyses. The sample sizes were *n* = 9 litters for NP-MD-8, LP-MD-8, and LP-Alc-8 groups; *n* = 8 litters for NP-Alc-8 group. *P* values before and after multiple testing adjustment using Benjamini-Hochberg method are presented

*Statistical significance at *P* < 0.05

**Table 6:** Correlation of the anthropometric measurements of the fetuses from dams in Groups 1 and 3 with their placental weight and efficiency

	Correlation with placental weight			Correlation with placental efficiency		
	*R*	*P*_unadjusted_	*P*_BH-adjusted_	*R*	*P*_unadjusted_	*P*_BH-adjusted_
Males only						
Fetal body weight	0.15	0.20	0.32	0.73	< 0.001	< 0.001*
Fetal brain-to-body weight ratio	0.05	0.65	0.74	− 0.55	< 0.001	< 0.001*
Fetal heart-to-body weight ratio	− 0.09	0.43	0.57	− 0.10	0.42	0.48
Fetal liver-to-body weight ratio	0.25	0.036	0.09	− 0.18	0.12	0.19
Fetal brain-to-liver weight ratio	− 0.23	0.046	0.09	0.01	0.95	0.95
Females only						
Fetal body weight	0.17	0.10	0.13	0.65	< 0.001	< 0.001*
Fetal brain-to-body weight ratio	− 0.21	0.036	0.072	− 0.33	0.001	0.002*
Fetal heart-to-body weight ratio	− 0.19	0.059	0.09	− 0.06	0.54	0.62
Fetal liver-to-body weight ratio	0.44	< 0.001	< 0.001*	− 0.19	0.064	0.10
Fetal brain-to-liver weight ratio	− 0.42	< 0.001	< 0.001*	0.03	0.79	0.79

Litters from all 4 treatments were combined and included in the analyses. The sample sizes were *n* = 9 litters for NP-MD-8, LP-MD-8, and LP-Alc-8 groups; *n* = 8 litters for NP-Alc-8 group. *P* values before and after multiple testing adjustment using Benjamini-Hochberg method are presented

*Statistical significance at *P* < 0.05

## Discussion

One major finding from our investigation is that placental weight and efficiency were the strongest indicators of fetal growth across multiple adverse stressors. Moreover, these placental predictors were sex-dependent. Specifically, placental efficiency predicted fetal outcomes for both sexes, whereas placental weight predicted outcomes only for the females, and these were conserved across the stressors examined in our study. Hence, our data suggest that fetal stress response is largely independent of the litter characteristics (e.g., litter size) and is instead driven primarily by the individual fetus and its sex. This observation holds until the stressor reaches a severity threshold that alters litter characteristics, as in the case of simultaneous exposure to multiple stressors.

For the stressors we examined, the placenta was a stronger predictor of fetal growth than was litter size. This likely reflects the ability of the placenta to adjust and adapt to the stressors. As the in utero sensor for the fetus [27, 28], the placenta generates compensatory responses to the adverse perturbations. These adaptations are both morphological and functional and include reduced thickness and increased surface area, fewer trophoblast cell death, increased blood vessel density, and an upregulation in the abundance or activity of nutrient transporters [39–41]. Gardebjer and colleagues [42] report that the placentas from pregnant rats, exposed to alcohol for a short duration around the periconceptional period, exhibit a greater glycogen cell area indicating glycogen accumulation, and a gene expression profile consistent with increased placental angiogenesis. Using a non-human primate model of alcohol exposure, Lo and colleagues [43] identified an adaptive response that involves changing the placenta’s growth trajectory and perfusion efficiency when the alcohol exposure occurred transiently during the first trimester. We speculate that similar mechanisms operate in placentas exposed to the mild alcohol stressor (Group 2).

When the stressor is of moderate severity, however, these placental adaptations may not be adequate in maintaining normal fetal growth. In these situations, the fetus must prioritize resources to maintain normal development of crucial organs, particularly the brain and heart, at the expense of less immediate needs, such as rapid body growth. The end outcome is that the placental blood carrying nutrients and oxygen is preferentially directed to address these priority needs, and thus, reducing the availability of these resources to other organs such as the fetal liver. Consequently, the fetal liver is small, the so-called “brain-sparing” phenomenon occurs, and the fetus exhibits asymmetric growth restriction [44, 45]. In support of this, fetuses that were exposed to alcohol for a longer gestational period (a moderately severe stressor) had higher fetal brain-to-body weight, heart-to-body weight, brain-to-liver weight, and lower liver-to-body weight ratios. In addition to these organ systems, placental blood redistribution may also have adverse impacts on fetal integumentary, musculoskeletal, renal, auditory, and vestibular systems, as suggested by an alcohol-dysregulated placental protein profile indicative of poor development of these organs [46]. Although this compensatory mechanism confers an immediate survival advantage to the fetus, it may have altered normal organ functions, thereby predisposing the offspring for metabolic or physiological dysfunctions in postnatal life [47].

Under severe insult, especially when maternal survival is also threatened, placentas do not have adequate resources to adapt and fully support the normal development of the fetus, putting the fetus at increased risk for intrauterine mortality. In such conditions, litter characteristics override the placental responses in becoming the first determinant of fetal outcomes. Specifically, the poorly developed fetuses will be terminated to reduce the intra-litter competition, allowing the allocation of the limited resources to support fetuses who are better developed, and thus, have a higher survival likelihood. We believe that this phenomenon contributes to the smaller litter size observed in dams exposed to both alcohol and protein insufficiency, who were likely having a severely stressful pregnancy. This strategy of selecting for long-term survivors likely operates in human pregnancy as well, albeit that they do not typically carry a large number of fetuses in each pregnancy like rodents do. This is supported by data indicating greater pregnancy losses during The Siege of Leningrad in 1942 [48], the Dutch famine of 1944–1945 [49], post-WWII food shortage in Germany [50], and the Chinese famine of 1959–1961 [51], when the pregnant mothers experienced a prolonged period of severe stress.

Our work also reveals that the capacity for placental adaptation to support fetal growth is sex-dependent. This phenomenon is not limited to maternal alcohol intake or protein insufficiency but is commonly reported in studies investigating other prenatal stressors including maternal dietary restriction, maternal asthma, preeclampsia, and gestational diabetes [52–57]. Although well-documented, the underlying basis for these sex-dependent placental responses remains unclear. The Trivers-Willard hypothesis states that this sex difference originates from their different reproductive strategies [58], wherein females, who have a higher chance of reproductive success than males, receive more maternal investments under adverse intrauterine conditions. Alternatively, it may be because male fetuses grow faster than female fetuses, and thus, employ a different strategy in response to adverse gestational insults [59, 60]. These hypotheses are not mutually exclusive.

Interestingly, the predictive effects of placental efficiency and placental weight on fetal growth in the presence of our stressors differed by sex. Placental efficiency, which is the ratio of fetal to placental weight, influenced fetal growth in a sex-independent fashion. This may be because placental efficiency relates to placental nutrient supply, which is determined by fetal demand based on its body growth [40]. Given that both sexes would similarly invest in supporting their own growth, the influence of placental efficiency may therefore be similar between the two sexes.

Conversely, placental weight influenced fetal growth in a sex-dependent manner, and it only predicted female fetal growth in our model. The work by Kalisch-Smith and colleagues [61] suggests that this relationship may originate from a different placental developmental trajectory in mid-gestation when the female placental development may lag behind that of the male placentas. Although the female placentas “catch up” by late gestation, this delay likely has some effects on the female fetal growth and may contribute to the observed predictive relationship in the females. Additionally, female placentas have more adaptive gene expression changes [55, 59] and greater glycogen and lipid stores [42, 62], suggesting that female fetuses have a greater ability to invest more resources in supporting placental development and placental nutrient reserves to protect themselves from additional insults. In contrast, the greater growth of the male fetuses requires a greater draw of resources, which may preclude them from investing and maintaining placental reserves while sustaining their rapid body growth. Their strategy works well if there are abundant resources in the intrauterine environment. Indeed, it may even reward them by enhancing their future reproductive success, because their greater body size is regarded as more fit, and enables them to outcompete their peers and attract female mating partners so that their genes can be passed on to a greater number of offspring [63, 64]. However, this risky strategy may backfire when the intrauterine insult is so severe, such as the co-exposure of maternal alcohol and protein insufficiency, that this emphasis on rapid growth greatly outstrips their placental nutrient reserves, leading to their death [60, 65]. Interestingly, May et al. [23] report a higher mortality risk among male children whose alcohol-abusing mothers also experienced protein insufficiency during their pregnancy [18]. Our preclinical study, which models the protein intake of these mothers, suggests that placental failure may be one explanation for the higher male mortality.

Using a mouse model of preconception paternal alcohol exposure, Bedi and colleagues [66] demonstrated that both male and female fetuses from the alcohol-exposed sires exhibit growth reduction similar in magnitude to those detected in the fetuses from our alcohol-exposed dams. Consistent with data from our mouse model of maternal gestational alcohol exposure, the fetal body reduction in their model was associated with the lower placental efficiency. However, whereas maternal drinking reduced only the female placental weight, and the placental weight predicted body weight only in the females, paternal drinking affected only the male placental weight and correlated with male fetal growth. These data point to an intriguing possibility that the sex-dependent influences of the placenta upon the fetus may also be determined by the parental origin of this alcohol insult.

Our study had several limitations. The oral gavage model employed in our study may have introduced additional stress, although we note this was experienced by dams in both the alcohol and maltodextrin exposure groups. We also acknowledge that alcohol exposure during the periconceptional and early gestational periods also has adverse consequences on the placenta and fetus. Our alcohol exposure windows did not include these gestational times, and the current work did not assess their additional impact. Finally, although rodents are multiparous, our data indicate that their responses to pregnancy stressors are consistent with those reported in humans. As such, our study findings are relevant to human pregnancy.

In summary, investigations using stressors that varied in timing and duration of alcohol exposure as well as maternal dietary protein intake demonstrate that the placenta is a significant determinant of fetal growth, and that each fetus alters its placental development, in a sex-dependent manner, to respond to the alcohol insult. Our data further suggest that these placental responses are unlikely stressor-specific but may be reflective of a more global response to gestational perturbation. These placental adaptations can accommodate fetal development until the perturbations are so severe that fetal demise is inevitable. This fetal stress response is largely sex-dependent and occurs at the individual fetus level and not at the litter level.

## Perspectives and significance

Not many studies in the field of FASD have investigated the interactive influences of maternal alcohol abuse and other secondary stressors upon fetal growth outcomes. Our study addresses this knowledge gap and provides important insights into the candidate mechanisms that may be responsible for the wide range of growth patterns observed in children affected with FASD. Because our findings identify the placenta as being one major determinant of fetal growth, molecular analyses of the placenta, such as the proteomic approach employed by Davis-Anderson and colleagues [46], are warranted to provide further understanding on how it contributes to the growth deficits accompanying these common stressors in human pregnancy.

## Supplementary information

**Additional file1: Table S1**. Effect of gestational alcohol exposure, a shorter alcohol exposure window, and maternal protein intake on maternal body weight and food intake during gestation. **Table S2**. Effect of gestational alcohol exposure, a shorter alcohol exposure window, and maternal protein intake on absolute fetal organ weight at E17.5.

## Data Availability

The datasets used and/or analyzed during the current study are available from the corresponding authors on reasonable request.
