# Supplementary material for: An interaction between fetal sex and placental weight and efficiency predicts intrauterine growth in response to maternal protein insufficiency and gestational exposure window in a mouse model of FASD
Source: Biol Sex Differ. 2020 Jul 20;11:40. doi: 10.1186/s13293-020-00320-9 (PMC7372829; doi:10.1186/s13293-020-00320-9)
Supplement: Supplementary file 1 — Additional file1: Table S1. Effect of gestational alcohol exposure, a shorter alcohol exposure window, and maternal protein intake on maternal body weight and food intake during gestation. Table S2. Effect of gestational alcohol exposure, a shorter alcohol exposure window, and maternal protein intake on absolute fetal organ weight at E17.5. [file 13293_2020_320_MOESM1_ESM.docx]

**Supplemental Table 1:**

Effect of gestational alcohol exposure, a shorter alcohol exposure window, and maternal protein intake on maternal body weight and food intake during gestation. ^a^

|  | **Group 1** | | **Group 2** | | **Group 3** | |
| --- | --- | --- | --- | --- | --- | --- |
|  | **NP-MD-8** | **NP-Alc-8** | **NP-MD-13** | **NP-Alc-13** | **LP-MD-8** | **LP-Alc-8** |
| Body weight at E0.5 (g) | 18.12 ± 0.25 | 17.88 ± 0.21 | 18.33 ± 0.20 | 17.72 ± 0.44 | 17.50 ± 0.56 | 17.25 ± 0.30 |
| Body weight on the first day of gavage (g) ^b^ | 20.47 ± 0.31 | 20.06 ± 0.23 | 26.30 ± 0.47 ^c^ | 25.37 ± 0.55 ^d^ | 20.22 ± 0.56 | 19.84 ± 0.30 |
| Body weight at E17.5 (g) | 31.03 ± 0.58 | 28.93 ± 1.17 | 31.30 ± 0.62 | 29.46 ± 0.72 | 30.41 ± 1.10 | 28.98 ± 0.69 |
| Gestational food intake (g) ^e^ | 55.54 ± 1.95 | 53.17 ± 1.80 | 52.29 ± 1.51 | 50.37 ± 1.55 | 50.36 ± 2.06 | 51.11 ± 2.06 |

^a^ Values are presented as means ± SEMs. The sample sizes were: n=9 dams for NP-MD-8, LP-MD-8, LP-Alc-8, and NP-MD-13 groups; n=8 dams for NP-Alc-8 group; n=10 dams for NP-Alc-13 group. Abbreviations: Alc, alcohol; E, embryonic day; LP, low protein; MD, maltodextrin; NP, normal protein. ^b^ For Groups 1 and 3, the first day of gavage was E8.5. For Group 2, the first day of gavage was E13.5. ^c^ Significantly different from NP-MD-8 group (*P* < 0.05). ^d^ Significantly different from NP-Alc-8 group (*P* < 0.05). ^e^ Gestational food intake refers to the amount of food consumed by the dams from E0.5 through E17.5.

**Supplemental Table 2:**

Effect of gestational alcohol exposure, a shorter alcohol exposure window, and maternal protein intake on absolute fetal organ weight at E17.5. ^a^

| **MALES** | **Group 1** | | **Group 2** | | **Group 3** | |
| --- | --- | --- | --- | --- | --- | --- |
|  | **NP-MD-8** | **NP-Alc-8** | **NP-MD-13** | **NP-Alc-13** | **LP-MD-8** | **LP-Alc-8** |
| Fetal brain weight (g) | 0.054 ± 0.001 | 0.054 ± 0.001 | 0.056 ± 0.001 | 0.055 ± 0.001 | 0.057 ± 0.002 | 0.055 ± 0.001 |
| Fetal heart weight (mg) | 3.714 ± 0.174 | 3.800 ± 0.185 | 4.016 ± 0.093 | 3.884 ± 0.116 | 3.642 ± 0.179 | 3.859 ± 0.204 |
| Fetal liver weight (g) | 0.044 ± 0.001 ^c,d^ | 0.028 ± 0.001 ^d,e^ | 0.040 ± 0.001 ^b^ | 0.034 ± 0.001 ^c^ | 0.043 ± 0.002 | 0.036 ± 0.001 |
| **FEMALES** | **Group 1** | | **Group 2** | | **Group 3** | |
|  | **NP-MD-8** | **NP-Alc-8** | **NP-MD-13** | **NP-Alc-13** | **LP-MD-8** | **LP-Alc-8** |
| Fetal brain weight (g) | 0.054 ± 0.001 | 0.054 ± 0.001 | 0.056 ± 0.001 | 0.051 ± 0.001 | 0.057 ± 0.001 | 0.054 ± 0.001 |
| Fetal heart weight (mg) | 3.589 ± 0.121 | 3.583 ± 0.181 | 4.266 ± 0.189 | 3.421 ± 0.159 | 3.650 ± 0.152 | 3.633 ± 0.175 |
| Fetal liver weight (g) | 0.046 ± 0.001 ^c,d^ | 0.027 ± 0.001 ^d,e^ | 0.039 ± 0.001 ^f^ | 0.037 ± 0.001 ^c^ | 0.043 ± 0.001 ^d^ | 0.037 ± 0.001 |

^a^ Values are presented as means ± SEMs. The sample sizes were: n=9 litters for NP-MD-8, LP-MD-8, LP-Alc-8, and NP-MD-13 groups; n=8 litters for NP-Alc-8 group; n=10 litters for NP-Alc-13 group. Abbreviations: Alc, alcohol; E, embryonic day; LP, low protein; MD, maltodextrin; NP, normal protein. ^b^ Significantly different from NP-Alc-13 group (*P* < 0.05). ^c^ Significantly different from NP-Alc-8 group (*P* < 0.05). ^d^ Significantly different from LP-Alc-8 group (*P* < 0.05). ^e^ Significantly different from LP-MD-8 group (*P* < 0.05). ^f^ Significantly different from NP-MD-8 group (*P* < 0.05).
